# Ten simple rules for dynamic causal modeling

**DOI:** 10.1016/j.neuroimage.2009.11.015

**Published:** 2010-02-15

**Authors:** K.E. Stephan, W.D. Penny, R.J. Moran, H.E.M. den Ouden, J. Daunizeau, K.J. Friston

**Affiliations:** a Laboratory for Social and Neural Systems Research, Institute for Empirical Research in Economics, University of Zurich, Blümlisalpstr. 10, 8006 Zurich, Switzerland; b Wellcome Trust Centre for Neuroimaging, Institute of Neurology, University College London, 12 Queen Square, London, WC1N 3BG, UK; c Donders Institute for Brain, Cognition and Behaviour, Centre for Cognitive Neuroimaging, P.O. Box 9101, 6500 HB Nijmegen, The Netherlands

**Keywords:** Effective connectivity, DCM, Bayesian model selection, BMS, Model evidence, Model comparison, Bayes factor, Nonlinear dynamics, fMRI, EEG, MEG, Synaptic plasticity

## Abstract

Dynamic causal modeling (DCM) is a generic Bayesian framework for inferring hidden neuronal states from measurements of brain activity. It provides posterior estimates of neurobiologically interpretable quantities such as the effective strength of synaptic connections among neuronal populations and their context-dependent modulation. DCM is increasingly used in the analysis of a wide range of neuroimaging and electrophysiological data. Given the relative complexity of DCM, compared to conventional analysis techniques, a good knowledge of its theoretical foundations is needed to avoid pitfalls in its application and interpretation of results. By providing good practice recommendations for DCM, in the form of ten simple rules, we hope that this article serves as a helpful tutorial for the growing community of DCM users.

## Introduction

Over the last two decades, neuroimaging analyses have become progressively refined and sophisticated. For example, there has been a trend away from the analysis of manually defined regions of interest to whole-brain analyses; from classical frequentist statistics to Bayesian hypothesis testing; and, most recently, efforts to construct mechanistic models of brain function. A representative of the latter is dynamic causal modeling (DCM), a generic approach for inferring hidden (unobserved) neuronal states from measured brain activity. DCM was introduced in 2003 for fMRI data (Friston et al., 2003) and made available as open-source software within the Statistical Parametric Mapping (SPM) software. The mathematical basis and implementation of DCM for fMRI have since been refined and extended repeatedly (Friston et al., 2007; Kiebel et al., 2007; Marreiros et al., 2008; Stephan et al., 2008, 2007c). Dynamic causal models (DCMs)^1^ have also been implemented for a range of measurement techniques other than fMRI, including electroencephalography (EEG), magnetoencephalography (MEG), and local field potentials (LFPs) obtained from invasive recordings in humans or animals, both in the time domain (Daunizeau et al., 2009b; David et al., 2006; Kiebel et al., 2006) and frequency domain (Chen et al., 2008; Moran et al., 2007, 2008, 2009; Penny et al., 2009).

DCMs are generative models of brain responses, which provide posterior estimates of neurobiologically interpretable quantities such as the effective strength of synaptic connections among neuronal populations and their context-dependent modulation. They are defined by five key features. First, DCMs are dynamic, using (linear or nonlinear) differential equations for describing (hidden) neuronal dynamics. Second, they are causal in the sense of control theory, that is, they describe how dynamics in one neuronal population cause dynamics in another and how these interactions are modulated by experimental manipulations or endogenous brain activity. Third, DCMs strive for neurophysiological interpretability. Fourth, they use a biophysically motivated and parameterized forward model to link the modeled neuronal dynamics to specific features of measured data (for example, regional hemodynamic time series in fMRI or spectral densities of electrophysiological data). Fifth, DCMs are Bayesian in all aspects. Each parameter is constrained by a prior distribution, which reflects empirical knowledge about the range of possible parameter values, principled considerations (e.g., certain parameters cannot have negative values) or a conservative attitude (e.g., “shrinkage” priors that express the assumption that coupling parameters are zero). Furthermore, Bayesian inversion not only provides posterior densities for each model parameter but also yields an approximation to the log model evidence, which is used to compare alternative DCMs of the same data.

Since their introduction in 2003, DCMs have gradually become part of mainstream neuroimaging analysis techniques. At the time of submitting this article (September 2009), the database PubMed listed more than 100 published papers on DCM. Its applications have concerned a wide range of domains in cognitive neuroscience, including language (Allen et al., 2008; Bitan et al., 2005; Leff et al., 2008; Noppeney et al., 2008; Schofield et al., 2009), motor processes (Eickhoff et al., 2005; Grefkes et al., 2008; Grol et al., 2007), vision and visual attention (Fairhall and Ishai, 2007; Haynes et al., 2005; Mechelli et al., 2003; Sonty et al., 2007), memory (Smith et al., 2006), perceptual decision making (Stephan et al., 2007b; Summerfield et al., 2006; Summerfield and Koechlin, 2008), and learning (den Ouden et al., 2009; Garrido et al., 2008, 2009). Given the relative complexity of DCM, compared to conventional analyses, many colleagues in the neuroimaging community have expressed an interest in a tutorial-like guide that addresses some of the most common questions about the theoretical foundations and empirical applications of DCM. This article represents an attempt to provide such a tutorial. It follows a recent tradition in the neuroimaging literature, inspired by the popular “10 simple rules” series in *PLoS Computational Biology* (Bourne, 2005), which has led to tutorial papers on, for example, voxel-based morphometry (Ridgway et al., 2008) and on reporting results from mass-univariate analyses (Poldrack et al., 2008).

In this article, we provide some generic “good practice” recommendations that address key conceptual and methodological issues in applying DCM to fMRI, EEG, MEG, or LFP measurements. Omitting any equations, we have tried to keep these recommendations as straightforward as possible. The suggestions made in this article should not be mistaken as dogmatic rules; instead, they are meant to provide guidelines for those users who are new to dynamic system theory, Bayesian statistics, and model selection procedures. Furthermore, some of the points below, such as the section on causality, are not concrete rules but outline the conceptual foundations of DCM. We anticipate that some of these guidelines and their underlying concepts may change over the forthcoming years, as both the theoretical foundations as well as the implementation of DCMs are progressively refined.

## Know what is “causal” about dynamic causal models

Causality in DCM is based on control theory (Friston, 2009): causal interactions among hidden state variables^2^ (e.g., specific aspects of neuronal population activity) are expressed by differential equations, which describe (i) how the present state of one neuronal population causes dynamics (i.e., rate of change) in another via synaptic connections and (ii) how these interactions change under the influence of external perturbations (i.e., experimental manipulations) or endogenous brain activity. The differential equations endow the system with memory such that future states are influenced by current states; the coupling parameters (rate constants) determine the speed of these influences. The ensuing coupling is influenced by where and when the system is subject to external perturbations; i.e., sensory inputs driving activity in specific neuronal populations or modulatory inputs that render the strength of coupling context-sensitive. In other words, causality in DCM does not only rely on temporal precedence but also takes into account when and where the system is perturbed by external influences.

An equivalent perspective is to interpret the state equation of a given DCM as encoding a particular causal structure–function relationship (Stephan, 2004). This is because the state equation of a given DCM prescribes explicitly how system dynamics arises from system structure: it specifies formally how neuronal state changes, induced by external inputs, propagate both in space (i.e., according to the system's connectivity structure) and in time (i.e., how current states influence future states). Therefore, changing the pattern of external inputs or the connectivity structure in a given DCM leads to different predictions about the spatiotemporal pattern of measured system responses. By simulating data from models with specified causal mechanisms, it is straightforward to assess whether, for a given level of observation noise, DCM is capable of correctly inferring these mechanisms. This has been done using both the same neuronal equations as in DCM (e.g., Stephan et al., 2008) and using independently designed large-scale biophysical models of spiking neurons (Lee et al., 2006). Perhaps even more convincingly, several animal studies using independent techniques such as invasive recordings and microdialysis demonstrated that DCM can successfully infer neuronal processes from BOLD responses and field potentials, respectively (David et al., 2008; Moran et al., 2008).

Critically, the hidden neuronal states give rise to noisy observations through a forward mapping (e.g., neurovascular coupling in fMRI). This transform is crucial for inferring causal interactions, particularly when it is nonlinear and may differ across brain regions, as is the case in fMRI (David et al., 2008; Stephan et al., 2004). Therefore, in contrast to Granger causality (Granger, 1969), causality in DCM does not describe interactions among the observations themselves. Instead, DCM aims to infer interactions among hidden neuronal states that cause noisy observations through a (possibly nonlinear and spatially variable) mapping.

It is noteworthy that inferring causal influences among neuronal populations does not necessarily require information about conduction delays. While conduction delays are explicitly represented in (and estimated by) DCMs for electrophysiological data (cf. David et al., 2006), axonal conduction delays in either inputs or inter-regional influences do not play a role in DCMs for fMRI. Due to considerable inter-regional variability in hemodynamic response latencies, fMRI data do not posses enough temporal information to enable estimation of inter-regional conduction delays, which are typically in the order of 10–20 ms; for simulations investigating such timing issues, see Friston et al. (2003). Instead, the differential latencies of the hemodynamic response are accommodated by region-specific biophysical parameters in the hemodynamic model (Friston et al., 2000; Stephan et al., 2004). Nevertheless, because it is not only sensitive to temporal order in signal but also to the spatiotemporal structure of inputs to the system, fairly subtle processes can be identified with DCM. For example, a recent rodent study showed that given fMRI data from a network of regions with epileptiform activity, DCM can infer where the seizure originated (David et al., 2008); this inference was verified by concurrent invasive electrophysiological recordings.

In summary, causality in DCM is embodied by the mathematical form of the differential state equations and does not just reflect temporal precedence but also accounts for the effects of external perturbations. By inverting an explicit forward model,^3^ DCM infers causal effects among hidden neuronal states that give rise to noisy measurements through a mapping that can be nonlinear and variable across the brain.

## Know your hypothesis and how to test it

DCM was designed to test hypotheses about the neuronal mechanisms that underlie experimental measurements of brain responses. In other words, DCM allows one to specify a generative model of measured brain data, which is a specific probabilistic mapping from experimentally controlled manipulations via neuronal dynamics to observed data.

**Fig. 1:**
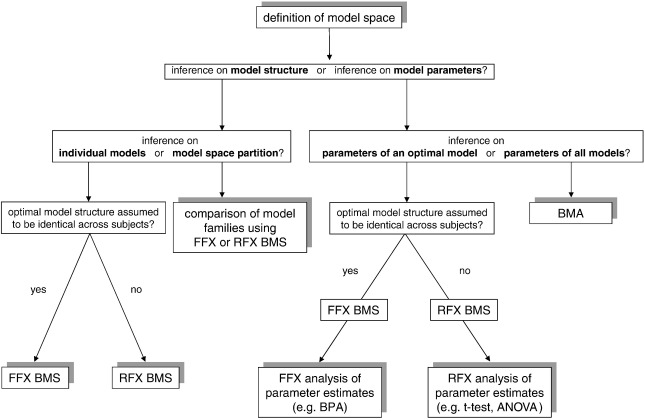
This schematic summarizes the typical sequence of analysis in DCM, depending on the question of interest. Abbreviations: FFX = fixed effects, RFX = random effects, BMS = Bayesian model selection, BPA = Bayesian parameter averaging, BMA = Bayesian model averaging, ANOVA = analysis of variance.

Importantly, two different types of inference can be obtained with DCM (Fig. 1). If one is not interested in any specific model parameter but in some aspect of model structure per se, then *inference on model space* is required. For example, one may wish to infer whether a particular neuronal system has a serial or parallel architecture, whether context-sensitive modulation of connectivity concerns forward or backward connections or whether the modulatory mechanism is linear or nonlinear. In other contexts, one may be interested in the neurophysiological mechanisms encoded by specific parameters in a given model; this requires *inference on model parameters*. For example, in a given model, one might want to infer whether a specific connection is more likely to exert an excitatory or an inhibitory effect on its target region. Prior to conducting a DCM study, one should clarify the type of inference required for the question at hand. This choice determines the sequences of data analysis steps as summarized in Fig. 1. Issues pertaining to inference on model space and parameters, respectively, are dealt with in more detail below.

In general, some scientific questions lend themselves more naturally to an analysis by DCM than others. A key feature of DCM is its dependence on experimental perturbations. Its state equations account for the influence of experimental manipulations on the system's dynamics: experimental conditions enter the model as inputs that either drive local responses or change connection strengths, respectively.^4^ For this reason, DCMs are usually only appropriate for explaining brain responses that are the consequence of specific experimental interventions. In contrast, data sets that were acquired in the absence of experimental control (e.g., resting state, sleep, hallucinations) are not suitable for DCM (a notable exception are DCMs with stochastic terms or inputs; cf. Daunizeau et al., 2009a; Moran et al. 2008). All other current DCMs with no experimentally controlled input predict nothing but a flat line. This is because, by design, they are based on dynamical systems with fixed point attractors. This can be regarded as a prior on brain dynamics that precludes exponential explosions of neuronal activity. (It is conceivable, however, that in the future, DCMs may be introduced that generate autonomous oscillations). This means that any time series chosen for subsequent DCM should, as a minimal requirement, show some relation to the experimental design. In fMRI, for example, this relation is usually established by an initial analysis using conventional statistical parametric mapping (SPM) on the basis of the general linear model (GLM). In fact, as is elaborated in more detail in point 8 below, DCM is typically used to compare different mechanistic explanations for specific activations detected by SPM. It is not unusual to ask questions about the interactions of areas with different response profiles; in this case, the areas included in a DCM are identified by different statistical contrasts in SPM. A typical example is the analysis of stimulus-by-task interactions in an area identified with the appropriate contrast. The response profile of such an area can be explained by task-dependent modulation of (i) one or several afferent connections from other areas identified by testing for a main effect of stimulus or of (ii) its self-connectivity (Allen et al., 2008; Stephan et al., 2007a).

Users of DCM (and other models of effective connectivity) sometimes worry that inference about the structure or parameters of a particular model may only be believable if the model as a whole shows a “reasonable” goodness of fit. Strictly speaking, this is an anecdotal concern, as has been noted by previous authors: “Unfortunately, there seems to be a belief that the ability to make inferences about changes in effective connectivity is compromised if the overall model does not fit the data adequately” (Protzner and McIntosh, 2006). These authors performed systematic simulation studies on the relation between model fit and inference about effective connectivity in the context of SEM and concluded that “one can detect differences in effective connectivity with SEM even when the overall model does not fit the data.” Having said this, it is usually the case that if the fits (accuracies) of competing models are poor, it is much more difficult to show that one model has greater evidence than another. In other words, a large Bayes factor indicates that the models compared fit the data sufficiently well to enable meaningful model comparison.

Some general points on the issue of model fit are worth highlighting here. There are many reasons why a perfectly reasonable model may fit a particular data set poorly, for example, independent observation noise. On the other hand, it is easy to construct complex models with excellent or even perfect fit, which are mechanistically meaningless and do not generalize (“overfitting”). This is why, as explained in the section entitled Use Bayesian model selection as a first step below, Bayesian model selection is a mandatory component of any DCM study: before making inference about any particular parameter, the model with the best balance between accuracy and complexity is selected from the set of all plausible alternative models (see section entitled Use Bayesian model selection as a first step). Furthermore, decisions on whether a particular model fit is “sufficient” or not depend on the number of data points. For example, the more data from the same process are available, the more likely it is that a *χ*^2^ test will reject the null hypothesis of no difference between predicted and observed covariance matrices (Bullmore et al., 2000). The opposite behavior is found when evaluating model fit on the basis of the coefficient of determination (*R*^2^ or “percent variance explained”): here, the longer the same process is observed, the more likely it is that any model will explain a significant amount of variance in the observed data. In summary, while any inference is always conditional on the model chosen, the validity of the model cannot be evaluated on the basis of its fit (cf. Pitt and Myung, 2002).

In contrast, establishing the validity of a given model requires additional external criteria. For DCM, *face validity* has been explored in terms of simulations (e.g., Friston et al., 2003; Stephan et al., 2008), *construct validity* has been established in relation to other models such as SEM (Penny et al., 2004b) and large-scale models of spiking neurons (Lee et al., 2006), and *predictive validity* has been addressed by verifying that DCM results fulfill predictions from independent experimental measures such as microdialysis (Moran et al., 2008) and invasive electrophysiological recordings (David et al., 2008).

In conclusion, when making statistical inferences about any particular mechanism underlying the data (as encoded by a combination of model parameters), model fit does not need to be considered explicitly; instead, it is an integral part of the model evidence that is optimized by BMS. The influence of model fit on inference enters vicariously by determining the precision of the estimates; this effect depends on which model parameters the inference is about (cf. standard equations for parameter estimates in a general linear model). This issue is no different for DCM than for standard regression models. In short, we cannot obtain *false inference* simply because of “poor” model fit.

Although inference can be about any aspect of model structure or any parameter in a DCM, the focus of DCM studies is typically on context-dependent changes in coupling. This corresponds to identifying physiological processes that change connection strengths at fast time scales ranging from milliseconds to seconds. DCM was developed for investigating these fast modulatory processes because they are critical for understanding the ubiquitous flexibility and context-sensitivity of neuronal circuits (Friston, 2002; McIntosh, 2000; Sherman and Guillery, 1998; Stephan, 2004). If specifying the anatomical source of these modulatory processes is not relevant for the scientific question of interest, a bilinear DCM is sufficient (Friston et al., 2003). Otherwise nonlinear DCM can be used, in which the strength of any given connection is allowed to depend on activity in remote neuronal populations (Stephan et al., 2008).^5^

To avoid erroneous interpretations of DCM results and accurately qualify the tested hypotheses, it is important to understand the neurophysiological mechanisms that underlie such fast modulatory processes. In brief, rapid changes of connection strength can result either from membrane excitability changes, synaptic plasticity, or a combination of both. For example, postsynaptic responses of ionotropic glutamatergic receptors are modulated by metabotropic receptors (Coutinho and Knopfel, 2002) and by receptors of various neuromodulatory transmitters (McCormick and Williamson, 1989). Alternatively, various forms of short-term synaptic plasticity can lead to fast changes in synaptic strength, e.g. synaptic depression and facilitation (Zucker and Regehr, 2002), NMDA- and dopamine-dependent phosphorylation of AMPA receptors (Chao et al., 2002; Wang et al., 2005), or dendritic spine motility (Holtmaat and Svoboda, 2009). All of these changes in synaptic strength can unfold within milliseconds to seconds.

In most instances, it is not important for the scientific question of interest to disambiguate whether modulatory processes identified with DCM reflect, at the neuronal level, changes in membrane excitability or synaptic plasticity. In fact, the two processes are often closely intertwined (Oh et al., 2009). If it is important to evaluate their relative contributions, it is necessary to adjust model structure and experimental design prospectively, using models that can represent both processes separately (Moran et al., 2008) or using experimental manipulations that are known to preferentially affect one of the two processes. An in-depth discussion of model-based inference about neuronal (patho)physiology with DCM is beyond the scope of the present article but is presented elsewhere (Stephan et al., in preparation).

In summary, DCM tests competing hypotheses about the neuronal mechanisms underlying experimental measurements of brain activity. Inference about putative mechanisms can either concern model structure or estimates of model parameters. DCMs are most appropriate for explaining brain responses that are the consequence of experimental interventions and typically focus on context-dependent changes in coupling that are mediated by fast changes in membrane excitability and/or connection strength. DCM is therefore a potentially useful approach for studying neuromodulation and synaptic plasticity, respectively, particularly when invasive methods are precluded for practical or ethical reasons.

## Use Bayesian model selection as a first step

As described above, DCM can rely on two different types of inference, *inference on model space* and *inference on parameter space* of any given model, respectively. Here, we emphasize that even when one is interested in inference on model parameters, a first step is usually Bayesian model selection (BMS). BMS is an established procedure in statistics that rests on computing (an approximation to) the model evidence *p*(*y*|*m*), i.e., the probability of the data *y*, given some model *m*.^6^ The model evidence, which can be considered the “holy grail” of model comparison, quantifies the properties of a good model; that is, that it explains the data as accurately as possible and, at the same time, has minimal complexity. Mathematical explanations of these properties can be found elsewhere (Friston et al., 2007; MacKay, 1992; Penny et al., 2004a; Stephan et al., 2009a). In BMS, models are usually compared via their Bayes factor, i.e., the ratio of their respective evidences (Kass and Raftery, 1995) or, equivalently, their difference in log-evidence (relative log-evidence).

Importantly, the model evidence is also a measure of generalizability (Pitt and Myung, 2002), i.e., how well the model generalizes across different data sets, and can thus be seen as an equivalent (but computationally more efficient) approach to procedures like cross-validation (MacKay, 1992). This may be easier to understand by noting that the model evidence is the likelihood of the data, having taken into account the natural variability of model parameters or, more formally, the prediction of data under random sampling from the prior densities of its parameters.

For inference on model space, BMS is sufficient, but it can be applied in different ways (Fig. 1). In brief, one can either identify a single optimal model, or one can choose a model space partitioning approach and compare sets or families of models that differ in one or several structural aspects (Penny et al., submitted for publication; Stephan et al., 2009a). Different options for using BMS, in the context of DCM, will be discussed in more detail below.

When inferring on model parameters, one needs to evaluate their estimated posterior densities. Critically, however, these posterior densities are conditional on the particular model chosen. For this reason, BMS is usually a requirement even when the hypothesis concerns values of model parameters and not model structure per se.^7^ Usually, one defines all plausible models in a first step, then uses BMS to select an optimal model from all alternatives, and finally proceeds to reporting posterior or conditional inferences about the parameters of this optimal model. This approach has been used by numerous studies in the recent past (e.g., Acs and Greenlee, 2008; Leff et al., 2008; Stephan et al., 2007b; Summerfield and Koechlin, 2008). For single-subject analyses, inference about any particular parameter (or about linear combinations of parameters) is straightforward; one can simply evaluate the posterior density of the parameter of interest, quantifying the probability that the value of the parameter is larger or smaller than some threshold (Friston et al., 2003). For multi-subject analyses, two options exist depending on whether one assumes that the parameters of interest are fixed effects in the population (FFX) or are themselves probabilistically distributed in the population (random effects, RFX). These issues are discussed in more depth in the section entitled Choose an appropriate method for group-level inference on parameters.

In summary, model selection is an essential component of any DCM study and is not normally omitted unless there is extremely strong *a priori* knowledge about the model. An important distinction is whether the hypothesis of interest concerns model structure per se or estimates of particular parameters in an optimal model. In the former case, BMS alone is sufficient to address the hypothesis, whereas in the latter case, a hierarchical or sequential procedure is necessary, where inference about particular parameters follows an initial model selection procedure.

## Motivate model space carefully

For any experimental observation, there exists, in principle, an infinite number of possible models that could explain the data. At first glance, this may appear a daunting state of affairs: how can one ever discover the “true” model, given an infinity of alternatives? It is helpful to remember that models are never true; by construction, they are meant to be helpful caricatures of complex phenomena, such that mechanisms underlying these phenomena can be tested. This insight is reflected in the well-known statement by Box and Draper (1987): “Essentially, all models are wrong, but some are useful” (p. 424). The purpose of model selection is to determine that model, from a set of plausible alternatives, which is most useful, i.e., represents the best balance between accuracy and complexity and thus affords maximal generalizability (Pitt and Myung, 2002).

The critical question in practice is how many plausible model alternatives exist; in other words, how large is the model space that must be searched? For small systems (i.e., networks or graphs with a small number of nodes), it is possible to investigate all possible connectivity architectures. However, when increasing the number of regions and inputs, evaluating all possible models becomes practically impossible very rapidly. Therefore, one of the first decisions, when conducting a DCM analysis, is to define the relevant model space, i.e., the set of models that are plausible, given prior knowledge about the system. This definition of the relevant model space should be as transparent and systematic as possible, and it should be described clearly in any article.

To ensure a clear definition and systematic exploration of model space, it is helpful to specify the important dimensions in model space and construct variations of models along these dimensions systematically. The definition of this space of plausible models could derive from principled considerations (e.g., combinatorial variations of a basic model structure) or could be informed by previous empirical studies using neuroimaging, electrophysiology, TMS, etc. in humans or animals. One option is to parameterize model space itself, where these parameters define a grid from which models can be sampled systematically. For example, models can be defined in terms of their priors, which could be a mathematical function of anatomical connectivity (e.g., tractography measures). Systematically varying the parameters of this function creates a model space (cf., Stephan et al., 2009b). Another approach is to create, under appropriate constraints, all possible combinations of components; for example, all possible combinations of modulatory inputs and/or driving inputs (for examples, see Kumar et al., 2007; Leff et al., 2008; Stephan et al., 2007b). A final option is to structure model space in a factorial fashion (Chen et al., 2009; Daunizeau et al., 2009b; Stephan et al., 2007c). The advantage of this approach is that it suggests a natural partitioning of model space into equally large subsets or families of models, which, as described below, can be compared collectively.

It may be useful to note at this stage that the definition of model space may or may not include variations concerning the regions contained by the model, depending on whether such variations change the data that are to be explained. In brief, for EEG/MEG data, model comparison can be used to decide about the number of regions, whereas for fMRI data, it cannot (see point 6 for details).

In summary, defining the dimensions of model space carefully is an important initial step in any DCM study. It prevents the modeler from getting lost in a space of infinite possibilities; it operationalizes the thinking about what constitutes plausible alternatives; and it is the basis for model space partitioning. This allows for powerful inferences about model structure, as described in the next section.

## Choose an appropriate method for group-level inference on model structure

Several options exist for performing BMS at the group level. As for all group analyses, a choice must be made between fixed-effects (FFX) and random-effects (RFX) analysis. In the FFX case, one assumes that the optimal model is the same for each subject in the population. This assumption is warranted when studying a basic physiological mechanism that is unlikely to vary across the subjects sampled. For example, FFX approaches to group-level BMS have been used to investigate the relation between anatomical connection probability and strength of functional coupling (Stephan et al., 2009b) and asymmetries between forward and backward connections in the visual system (Chen et al., 2009). Under the FFX assumption, a useful metric is the group Bayes factor (GBF, Stephan et al., 2007c), which expresses the evidence for one model relative to the evidence for another model, considering the group as a whole. The GBF has a simple definition: because Bayes factors are probability ratios, which are independent across subjects, the GBF is the product of individual Bayes factors. When comparing more than two models, it is more straightforward to report the group log-evidence for each model, which is just the sum of log-evidences across subjects. In practice, the simplest and most informative way to report the (group) log-evidence is to show a bar chart of log-evidences over models, after subtracting the log-evidence for the model with the least evidence (e.g., Garrido et al., 2009; Stephan et al., 2009b). Readers can then tell at a glance which model(s) had the greatest evidence and whether the differences were quantitatively important. Usually, a difference in log-evidence of three is taken as strong evidence (Kass and Raftery, 1995) because the corresponding Bayes factor of exp(3) is about twenty (cf. the *p* < 0.05 criterion often employed in classical inference).

However, assuming that the optimal model structure is a fixed effect in the population may not be appropriate. For example, when investigating pathophysiological mechanisms in a spectrum disease or when dealing with cognitive tasks that can be performed with various cognitive strategies (and thus implemented neuronally in different ways), it is more appropriate to adopt a random-effects (RFX) BMS procedure. An early suggestion for a simple RFX index was the positive evidence ratio (PER, Stephan et al., 2007c). This is simply the ratio of how many subjects showed positive evidence^8^ for one model relative to another. The PER can be considered a special case of a general and fully probabilistic RFX procedure for BMS. This generalized method uses variational Bayes (VB) to estimate the posterior probabilities of competing models, given data from a population of subjects (Stephan et al., 2009a). Based on this, one can compute how likely it is that a specific model generated the data of a randomly chosen subject (i.e., the expected posterior model probability) as well as the exceedance probability that one model is more likely than any other model, given the group data. Evaluations based on synthetic and empirical data have shown that this method is accurate and robust; in contrast to FFX analyses, outliers have very little impact on the results (Stephan et al., 2009a). With the availability of this method in SPM8, the use of the PER should be abandoned.

In addition to comparing specific models one can also compare subsets (families) of models, which result from a partition of model space. For example, BMS can be used to quantify the probability that the presence versus the absence of a particular connection improves model performance, regardless of any other differences among the models considered. This type of inference rests on comparing two (or more) subsets of model space, pooling information over models in each subset. This effectively removes uncertainty about any aspect of model structure, other than the attribute of interest (which defines the partition of model space). This approach currently represents the method of choice when the hypothesis to be tested concerns model structure and not any specific parameter. This sort of inference is available for both fixed and random-effects group models (Penny et al., submitted for publication; Stephan et al., 2009a).^9^^,^^10^

In summary, FFX BMS assumes that the optimal model is identical across the population and uses the GBF or group log-evidence to quantify the relative goodness of models. In contrast, RFX BMS accounts for heterogeneity of model structure across subjects and yields posterior model probabilities and exceedance probabilities. In either case, model space partitioning and subsequent comparison of model families (family-level inference) should be considered when the hypothesis of interest concerns model structure and not any particular model parameter.

## Know what you can and cannot do with Bayesian model selection

BMS based on (approximations to) the log-evidence is a principled and computationally efficient method for determining an optimal model from a set of competing alternatives, given some data.^11^ For example, one can compare DCMs that differ in terms of which inputs affect the system, where these inputs enter, whether mechanisms are linear or nonlinear, which anatomical connections exist, or which priors are best (Acs and Greenlee, 2008; Chen et al., 2009; Garrido et al., 2008; Stephan et al., 2008, 2009b, 2007c). In other neuroimaging domains, BMS is not just being used for DCMs but is also routinely employed to decide between alternative source reconstructions procedures for EEG/MEG data (e.g., Friston et al., 2008; Henson et al., 2009; Kiebel et al., 2008). BMS has also been frequently used in machine learning (Chu et al., 2007; MacKay, 1992; Penny and Roberts, 1999) and neuroeconomics, for example, to distinguish between competing models of learning and decision making (Brodersen et al., 2008; den Ouden et al., submitted for publication; Hampton et al., 2006). There are, however, some caveats in using BMS and interpreting its results in the context of DCM that a user should be aware of.

A mathematically trivial but practically important issue is that the model evidence is defined with respect to one particular data set. This means that BMS cannot be applied to models that are fitted to different data. Specifically, in DCM for fMRI, one cannot compare models with different numbers of regions, because changing the regions changes the data (this is a consequence of the data reduction used in DCM for fMRI, in which only data from regions of interest are included). In the case of DCM for MEG/EEG, however, the data to which the model is fitted (i.e., the spatiotemporal distribution of electric potentials or magnetic fields at the sensor level) is always the same, regardless of how many regions (sources) are included in the model. In fact, DCM can be considered a source reconstruction approach that exploits information about coupling among sources and can be used to determine the most likely number and deployment of sources (Kiebel et al., 2006).

A more complicated issue, which frequently occurs in exchanges with reviewers, is that any measure of model goodness is relative, not absolute. This is true for any approximation to the model evidence (e.g., the Akaike and Bayesian information criteria, or free energy) and the ensuing Bayes factor. It also applies to log-odds ratios and other frequentist statistics like the classical coefficient of determination (*R*^2^), which, in the context of linear models, is often interpreted as the proportion of variance explained by the model. Although *R*^2^ may appear, at first glance, like an absolute goodness of fit index, it is simply the result of a model comparison. Mathematically, this can be seen easily from the generalized definition of *R*^2^ (e.g., Nagelkerke, 1991), which reveals that *R*^2^ is always determined relative to an (implicit) null model. For example, in the context of linear models, this null model is extremely simple, consisting of a constant (or intercept) only. See the section entitled Know your hypothesis and how to test it for a discussion on why measures such as *R*^2^ are not useful for evaluating DCMs.

The relative nature of inference obtained by BMS also pertains to the RFX BMS procedure described above: here, the posterior model probabilities are a function of the set of models considered. In other words, these estimates can change when reducing or extending model space. Although mathematically this behavior is perfectly reasonable (for details, see Stephan et al., 2009a), it can produce seemingly counterintuitive results, when sequentially performing BMS on parts of model space that have a nested or overlapping relationship (Penny et al., submitted for publication). To prevent problems of interpretation, such sequential tests should be avoided; instead, one should perform BMS on the entire space of plausible alternative models in one step.

In summary, it is important to keep in mind that any result obtained by BMS, or indeed any other model selection procedure, expresses a relative statement about model goodness that is conditional on the model space considered. Again, this highlights the importance of a careful and principled definition of model space (cf. section entitled Motivate model space carefully).

## Choose an appropriate method for group-level inference on parameters

When analyzing parameter estimates across the group, the same decision must be made as for group-level BMS (see section entitled Motivate model space carefully). That is, the modeler needs to decide whether the mechanisms encoded by the model parameters of interest are likely to exist as fixed or random effects in the population. If they can be considered fixed effects, e.g., when dealing with low-level physiological properties, several alternative procedures exist. One commonly employed method is Bayesian parameter averaging (BPA). This effectively computes a joint posterior density for the entire group by combining the individual posterior densities, treating the posterior from one subject as the prior for the next (Garrido et al., 2007; Neumann and Lohmann, 2003). The mathematical advantage of this commutative procedure is threefold. First, it accounts for posterior covariances among the parameters; second, under Gaussian assumptions about the posterior, it is extremely easy and efficient to compute; and finally, it produces a single posterior density for the entire group that can be used for Bayesian inference (cf. (Acs and Greenlee, 2008)). However, BPA also has some disadvantages (Kasess et al., 2010). One potential problem is that the posterior covariances can make the posterior estimates behave in a counterintuitive way, even when they are mathematically perfectly sensible. This can become particularly severe for data with a high signal-to-noise ratio where, in the presence of pronounced posterior covariances, the Bayesian average can deviate substantially from the mean of the maximum a posteriori (MAP) estimates across subjects. Other fixed-effects methods do not suffer from this particular problem, although they have other restrictions; for details see Kasess et al. (2010). These alternatives include the univariate variant of BPA and simple temporal averaging of the subjects' time series as a pre-processing step (which is only possible if the stimulus timing is identical across subjects).

If a fixed-effects analysis is not appropriate and one thinks that the parameters are random effects in the population (e.g., task-induced changes in connection strengths in cognitive paradigms), a simple solution exists. This approach consists of entering the subject-specific MAP estimates into a second-level frequentist test (e.g., a *t*-test or ANOVA). This procedure is simple and robust and has found widespread application. It is conceptually identical to the summary statistic approach used in conventional SPM analyses, only here the summary statistic is a maximum *a posteriori* estimate (as opposed to a maximum likelihood estimate).

An alternative approach is Bayesian model averaging (BMA) (Hoeting et al., 1999; Penny et al., submitted for publication).^12^ This approach abandons the dependence of parameter inference on the particular model chosen. Instead, it uses the entire model space considered (or an optimal family of models, Penny et al., submitted for publication) and computes weighted averages of each model parameter, where the weighting is given by the posterior probability for each model. It represents a useful alternative, particularly when none of the models (or model subspaces) considered clearly outperforms all others. In this case, one can take the uncertainty about model structure into account by pooling information across all models in a weighted fashion, as described above. BMA is also a promising method for comparing parameter estimates across groups (e.g., patients versus controls) for cases where BMS indicated a group difference with regard to the optimal model. If one does not wish to restrict the group comparison to inference on model structure, one can use BMA to compute the average parameter estimates across all models and then statistically compare these averages between the two groups.

Finally, it should be mentioned that testing hypotheses about multiple parameters requires a correction for multiple comparisons (unless a single contrast, i.e., linear combination of these parameters, is examined). A Bonferroni procedure would be the simplest way to do this, even though this is conservative in the presence of posterior dependencies among the parameters tested.

In summary, there are two principled approaches to making group-level inferences about specific model parameters (Fig. 1). The first approach entails finding an optimal model in an initial BMS step and then examining the parameter estimates across the group, using either FFX or RFX methods. A second possible approach is BMA that computes a weighted average of each model parameter, where the weighting is determined by the posterior probability of each model.

## Optimize experimental design and data acquisition

A frequently asked question is what experimental designs and acquisition techniques are optimal for DCM analyses. The answer may differ somewhat for hemodynamic and electrophysiological measurements. As a rule of thumb, however, the same optimization strategies for design and data acquisition that apply to conventional generative models of brain activity (e.g., the General Linear Model) also apply to DCM. This is because DCM aims to explain the same phenomena, i.e., regional brain responses, as other generative models, and is thus subject to similar constraints concerning experimental design and data acquisition. The critical difference lies in the mechanisms the models have at their disposal to explain observed data. DCM not only takes into account the direct influence of experimentally controlled variables on regional activity (as the GLM) but considers interactions among neuronal populations and how these interactions are modulated by experimental perturbations. DCM is thus a very generic model and includes other generative models of brain responses as special cases. For example, the GLM for fMRI data and source reconstruction methods for EEG/MEG can be thought of as special cases of DCMs (for details, see Daunizeau et al., 2009b; Kiebel et al., 2006; Stephan et al., 2007a). Similarly, conventional models of effective connectivity, such as Structural Equation Modeling (SEM; Bullmore et al., 2000; Horwitz et al., 1999; McIntosh and Gonzalez-Lima, 1994) can be understood as a special case of DCM where states are assumed to have reached equilibrium at the point of observation (Friston et al., 2003).

In terms of experimental design, DCM is especially useful for factorial designs whose levels can be interpreted as inducing driving effects (such as sensory stimulation) and modulatory effects (such as learning or attention), respectively. Factorial designs are particularly attractive because they naturally embody the notion of interactions among experimental manipulations and thus context-sensitive neuronal responses, which are the typical explanatory target of a DCM study (see section entitled Know your hypothesis and how to test it). For example, if a conventional SPM analysis indicates a significant interaction between one experimental factor related to sensory stimulation and another factor of a more “modulatory” character (e.g., task demands, attention or learning), one may wish to understand how this interaction or context-sensitive response arises. This can be modeled straightforwardly using DCM with connections that convey stimulus-specific information, but are under the control of modulatory influences. With DCM and BMS one could investigate, for example, which of the many afferents to the target area are subject to modulatory inputs, and whether a specific neuronal population constitutes a likely anatomical source of these modulatory inputs (this requires nonlinear DCM, Stephan et al., 2008). Examples of this can be found in several papers (e.g., Heim et al., 2009; Stephan et al., 2007a). Another attractive option for experimental design is to focus on processes that (i) are known to induce synaptic plasticity, and thus changes in effective connectivity and (ii) have a known (or assumed) parametric form. A prototypical example fulfilling both criteria is learning. Here, DCM and BMS can be used to investigate which of several competing learning models best explain changes in connection strength in neuronal circuits involved in learning and which particular connections exhibit synaptic plasticity and thus contribute to learning (for examples, see den Ouden et al., 2009, submitted for publication).

Concerning data acquisition, two issues are worth highlighting in relation to DCM for fMRI. Due to the multi-slice acquisition of fMRI data, regional time series are sampled at different times relative to scan onset, and these timing differences represent a potential confound. Simulations indicated that timing differences up to about a second were tolerable and did not lead to significant deviations of parameter estimates from their true values (because these inaccuracies could be explained away by the hemodynamic model; Friston et al., 2003). As a consequence, a common recommendation used to be to restrict TA (time to acquisition)^13^ to 2 s or less and use the middle slice (in time) as reference for defining the inputs. This limitation was overcome by extending DCM for fMRI with a model of slice-specific sampling times (Kiebel et al., 2007). This extended model accounts for the times at which regional sampled were sampled and adjusts its predicted hemodynamic output accordingly.^14^

A second important issue in relation to data acquisition in fMRI concerns the order of acquired slices. For DCM, and other models of effective connectivity, a continuous acquisition scheme (with appropriate inter-slice gap) is advantageous, when compared to interleaved acquisition. In the latter scheme, any interpolation across neighboring slices (e.g., during realignment or spatial normalization) or extraction of representative time series from voxels across more than one slice, leads to the mixing of time series that were acquired at different times. This is suboptimal and should be avoided.

Concerning the application of DCM to EEG/MEG data, it is worth mentioning that the only strict requirement is to record the electrode positions on the scalp, as well as the usual positioning landmarks (left ear, right ear, and nasion fiducials). These are required to model the spatial expression of neuronal activity as measured electromagnetic fields at the scalp level. Although it is possible, in principle, to invert a DCM given data from a single electrode, model inversion is facilitated by a fine spatial sampling on the scalp.

Beyond data acquisition, it is important to decide which aspects of the data are of interest, i.e., feature selection. For example, in fMRI hundreds of thousands of voxel time series are acquired, resulting in a huge spatiotemporal data matrix. To reduce complexity and allow for meaningful inference, DCM for fMRI requires that one summarizes the distributed responses observed across the brain by selecting a few key regions involved in the process of interest. In defining these regions, one is implicitly specifying a structural model, which represents a data reduction or feature selection. From another perspective, this structural model is a parsimonious representation of other possible models, e.g., models with additional intermediate or relay regions. To reduce complexity, it is often possible to analyze a sub-network of key regions involved in a particular task. This is particularly straightforward when dealing with sensory “processing streams” where relay regions can be omitted (cf. Grol et al., 2007) because the coupling parameters represent the effective connectivity among regions, and this influence can be mediated polysynaptically (Friston, 1994). Also, one can replace endogenous inputs from a sub-network one is not interested in with exogenous (driving) inputs that approximate the influence from this sub-network. For example, one can replace both sensory and cognitive sub-networks with inputs representing the sensory stimuli (Heim et al., 2009; Smith et al., 2006) and the cognitive process (den Ouden et al., submitted for publication; Stephan et al., 2008), respectively. In a next step, it is then possible to optimize this substitute for the omitted sub-network by comparing different spatial distribution of the exogenous inputs using BMS (cf., Leff et al., 2008). Given these possibilities for reduction of model complexity, it is perhaps not surprising that some of the most powerful applications of DCM have used networks with as few as two or three nodes. Note that, as explained in the section entitled Know what you can and cannot do with Bayesian model selection, in DCM for fMRI (but not in DCMs for electrophysiological data) BMS cannot be used to decide whether a region should be included or excluded because the model evidence is defined with regard to a specific data set and changing the regions changes the data.

In DCM for fMRI, once the regions have been selected, it is necessary to obtain a summary of their activity. For fMRI, this is usually accessed through the principal eigenvariate of the region of interest. This is just the first principal component of the local multivariate time series (over all voxels in the region). This procedure has advantages over other summary indices like the mean. For example, when dealing with functionally heterogeneous regions, it guarantees that positive and negative responses do not cancel in extracting a summary time series across voxels (Friston et al., 2006). In group studies, one wants to ensure that the same regional features are selected from subject to subject. The best way to do this is to operationally define the region in each subject by functional and anatomical criteria (see section entitled Use anatomical information and computational models to refine your DCMs) and then use the principal eigenvariate centered on each subject-specific region. This regional definition is usually based on the functional specialization revealed by a conventional SPM analysis (i.e., a maximum of an SPM testing for that region's responses).

Note that there is no circularity in using the same data to define regions by SPM and analyze their interactions with DCM. This is because, in contrast to SPM, the purpose of DCM is not to test whether any of these regions shows an experimental effect. Instead, as explained above, DCM serves to compare different hypotheses about the mechanisms (in terms of neuronal coupling) that underlie the regional responses detected in conventional analyses. For example, if SPM indicates that an experimental manipulation significantly increases the activity of a particular region, there are numerous possible explanations, which can be disambiguated by DCM. The observed increase in activity might (i) reflect the downstream consequence of a context-sensitive process elsewhere in the network that is conveyed via endogenous connections in a context-independent manner, (ii) result from changes in intra-regional inhibition (i.e., a modulation of the self-connection by the experimental factor), or (iii) arise from a modulation of one or several afferent connections by the experimental factor. Indeed, it would be nonsensical to ask this question of regional responses that did not show experimental effects. Generally, one should use the most revealing *t*- or *F*-contrast for each region, to identify the local maxima in subject-specific SPMs that are nearest to the maximum in the group SPM. One can then take the principal eigenvariate in a local region of interest that is centered on the subject-specific maxima and is ideally informed by additional anatomical criteria (cf. section entitled Use anatomical information and computational models to refine your DCMs). Future refinements of this procedure include augmenting DCMs with an explicit spatial model of regional responses (Woolrich et al., 2009). Alternative approaches can be based on anatomically defined regions of interest where the shape and form of the region depends on the spatial precision of the data. Irrespective of the method for summarizing regional activity, it should be described and motivated clearly and simply (cf. section entitled Report the modeling approach and results in detail).

## Use anatomical information and computational models to refine your DCMs

Both knowledge about anatomical structure and computational processes of the system of interest can help to optimize a DCM of that system. For example, neuroanatomical atlases can provide useful constraints for defining the regions included in a DCM. This is particularly relevant for multi-subject studies, which face the challenge of inter-individual variations in the exact location of a given brain area. Here, probabilistic cytoarchitectonic atlases, such as the anatomy toolbox in SPM (Eickhoff et al., 2005), can provide anatomical constraints that complement functional criteria (see section entitled Optimize experimental design and data acquisition) in choosing regional time series; for concrete examples, see Heim et al. (2009) and Stephan et al. (2007b).

The second useful source of anatomical information concerns the anatomical connectivity between regions of interest. Many previous studies of effective connectivity have used information from invasive tract tracing studies in the Macaque monkey to inform the structure of their models. These data are of high resolution and large quantity but entail potential inter-species differences. Human tractography studies, based on diffusion weighted imaging, do not suffer from this problem, but provide less detailed and non-directional information. Nevertheless, they can provide important structural constraints for defining DCMs. For example, anatomical connectivity information from probabilistic tractography studies can be formally integrated into DCMs in terms of anatomically informed priors (Stephan et al., 2009b). This approach rests on the fact that the deployment of anatomical connections constrains effective connectivity but does not fully determine it because synaptic connections can be expressed functionally in a dynamic and context-sensitive fashion (Breakspear, 2004; Friston, 2002; McIntosh, 2000; Stephan et al., 2008). A useful corollary is to define connection-specific priors such that the higher the likelihood of a given connection existing anatomically, the larger the prior variance of the corresponding coupling parameter in DCM; hence making it easier for the parameter to deviate from zero (in either direction) and represent a strong effective connection. Incorporating anatomical information in this way can improve the evidence of DCMs (Stephan et al., 2009b). Therefore, when available, DCMs can be informed by tractography data; the benefit of such anatomical constraints can then be assessed using BMS.

Prior knowledge about the nature of the computations involved in an experimentally induced cognitive process can also help to refine DCMs. For example, if an established computational model exists that predicts the temporal evolution of some cognitive variables, then any trial-by-trial values can enter a DCM, either as driving or as modulatory inputs that change connection strengths (Stephan, 2004). This is an attractive option in the context of learning, since this is the paradigmatic experimental manipulation to induce synaptic plasticity and therefore changes in effective connectivity. For example, several theories of learning agree on the notion that synaptic plasticity should be a function of prediction error (Friston, 2005; Montague et al., 1996; Schultz and Dickinson, 2000). This notion can be tested by augmenting DCMs with computational learning models (such as Rescorla-Wagner, temporal difference learning or Bayesian learning models), which describe the evolution of trial-by-trial prediction errors. These trial-wise prediction error estimates can be used as modulatory inputs in DCM, controlling changes in connection strength during learning (den Ouden et al., 2009). In a further step of refinement, nonlinear DCM can be used to specify the source of these modulatory influences anatomically. For example, a study of audio-visual associative learning showed that prediction error activity in the putamen, as modeled by a Bayesian learner, exerted a nonlinear modulatory influence on visuomotor connections, thus gating transfer of sensory information about unexpected stimuli (den Ouden et al., submitted for publication).

In summary, whenever available, *a priori* information about anatomy and computation should be used for refining DCMs. When anatomical or computational quantities are used as described above, one is effectively performing a form of multimodal integration that uses functional, anatomical as well as behavioral data. This confluence of computational and neurophysiological modeling techniques may prove particularly fruitful for a mechanistic understanding of neuronal systems.

## Report the modeling approach and results in detail

Although the principles of DCM are generic, understanding the structure of a specific model (or model space) built by someone else can be difficult, unless it is communicated very clearly. When reporting the results of the DCM study, it is helpful to include as many organizing details as possible; e.g., describe your thinking behind the construction of model space and why you included some attributes and not others. It is these choices that define the question you wish to address. This will help others to understand exactly what you have done and to replicate your results. The information that is required concerns all stages of the modeling process. For example, one should report the following:

– the anatomical and/or functional criteria used to define regions of interest and their summary time series;
– the resulting coordinates of regions and their consistency across subjects;
– how model space was motivated by the hypothesis;
– how families of models were defined, when using model space partitioning;
– the BMS procedure and approximation to the log-evidence used;
– BMS results for all models considered (e.g., by tabulating or plotting log-evidences, posterior probabilities, or exceedance probabilities for each model); and
– the parameters of the selected model (or family) in terms of the MAP estimates, or inference using the classical summary statistic approach or BPA.

Of course, it is not always necessary (or desirable) to include all of this information in the main text of an article; some of the information above can be included in the supplementary material or summarized in graphs and tables.

## Summary

DCM is a generic and powerful method for inferring causal mechanisms in systems, whose dynamics are observed indirectly. Following considerable success in its application to many domains of cognitive neuroscience and neurophysiology, the use of DCM is becoming increasingly widespread. However, given that DCM is a nontrivial technique for nonlinear system identification that does not afford “off the shelf” applications, knowledge of its conceptual and mathematical foundations is mandatory. This article has outlined procedures for good practice, focusing on what we perceive as the most relevant and generic issues. Clearly, there are more methodological aspects to DCM than can be addressed in a tutorial paper, and the interested reader is referred to the primary literature we have referenced. We hope that the ten simple rules described above will be helpful for the growing community of DCM users.

## Footnotes

1 We use the acronym DCM both to refer to the general approach (dynamic causal modeling) and to refer to the instantiation of a specific dynamic causal model.

2 The term “hidden state variables” refers to time-varying properties of systems that cannot be observed directly.

3 A forward model describes how a specific hidden state translates into a measurement. The “inversion” (fitting or solution) of a model describes the opposite process, i.e., to estimate the hidden state given the measurement.

4 For electrophysiological data, the driving inputs are parameterized themselves (e.g., in DCM for evoked responses; David et al. 2006) and can include stochastic inputs (e.g., white and pink noise in DCM for steady-state responses; Moran et al. 2009) that represent neuronal background activity not included in the model.

5 In bilinear DCM, the modulation of effective connectivity results from an interaction between neuronal activity in one region and experimentally controlled inputs; this context-dependent change in connectivity is additive. In contrast, in nonlinear DCM, changes in connectivity result from the interaction of neuronal activity in two different regions; this modulation is of a multiplicative nature.

6 For numerical reasons, it is often more convenient to use the log-evidence as an index of model goodness. This is equivalent because the logarithm is a strictly monotonic function, i.e., maximizing the log-evidence also maximizes the evidence.

7 In some (albeit rare) instances, one may have very strong *a priori* knowledge about the structure of the system of interest and can proceed directly to inference on parameters.

8 Following the widely used classification by Kass and Raftery (1995), “positive” evidence for one model versus another exists if the Bayes factor is larger than three.

9 At the time of writing this article, family-level inference based on model space partitioning is not yet available via the graphical user interface in SPM8. Instead, users can implement it by directly calling the SPM routine spm_compare_families.

10 The VB implementation of the random-effects BMS method is only applicable when the model families contain an equal numbers of models. When comparing model families of unequal size, a Monte Carlo Markov Chain (MCMC) method based on Gibbs sampling must be used instead (Penny et al., submitted for publication).

11 Computational efficiency of model selection procedures is becoming an increasingly important issue since the model space that is explored by standard neuroimaging studies is continually increasing. For large model sets comprising hundreds or thousands of models, alternative procedures based on sampling, e.g., cross-validation, become prohibitively expensive; cf., MacKay (1992).

12 This approach can be used both for single subjects and for groups; see Penny et al. (submitted for publication). An implementation of this procedure will be available in forthcoming SPM versions.

13 This is the time required to obtain one brain volume.

14 TA is usually equivalent to TR (time to repetition) unless the fMRI acquisition sequence includes a delay between subsequent scans, e.g., in sparse sampling schemes for auditory tasks. The duration of TR has no impact on DCM, and sparsely sampled fMRI data pose no difficulty for DCM analyses; c.f., Kumar et al. (2007).
